# Barrier-free, open-top microfluidic chip for generating two distinct, interconnected 3D microvascular networks

**DOI:** 10.1038/s41598-024-74493-3

**Published:** 2024-10-02

**Authors:** Alma Yrjänäinen, Elina Mesiä, Ella Lampela, Joose Kreutzer, Jorma Vihinen, Kaisa Tornberg, Hanna Vuorenpää, Susanna Miettinen, Pasi Kallio, Antti-Juhana Mäki

**Affiliations:** 1 Adult Stem Cell Research Group, Faculty of Medicine and Health Technology, Tampere University, Tampere, Pirkanmaa, Finland; 2 Tays Research Services, Wellbeing Services County of Pirkanmaa, Tampere University Hospital, Tampere, Pirkanmaa, Finland; 3 Micro- and Nanosystems Research Group, Faculty of Medicine and Health Technology, Tampere University, Tampere, Pirkanmaa, Finland; 4 Faculty of Engineering and Natural Sciences, Tampere University, Tampere, Pirkanmaa, Finland

**Keywords:** Organ-on-Chip, Microphysiological systems, Vascularization, Interstitial flow, 3D cell culture, Biomedical engineering, Biological models, Angiogenesis

## Abstract

Developing microphysiological cell culture platforms with a three-dimensional (3D) microenvironment has been a significant advancement from traditional monolayer cultures. Still, most of the current microphysiological platforms are limited in closed designs, i.e. are not accessible after 3D cell culture loading. Here, we report an open-top microfluidic chip which enables the generation of two sequentially loaded 3D cell cultures without physical barriers restricting the nurture, gas exchange and cellular communication. As a proof-of-concept, we demonstrated the formation of two 3D vasculatures, one in the upper and the other in the lower compartment, under three distinct flow conditions: *asymmetric side-to-center*, *symmetric side-to-center* and *symmetric center-to-side*. We used computational modelling to characterize initial flow pressures in cell culture compartments. We showed prominent vessel formation and branched vasculatures in upper and lower cell culture compartments with interconnecting, lumenized vessels with in vivo-relevant diameter in all flow conditions. With advanced image processing, we quantified and compared the overall vascular network volume and the total length formed in *asymmetric side-to-center*, *symmetric side-to-center* and *symmetric center-to-side* flow conditions. Our results indicate that the developed chip can house two distinct 3D cell cultures with merging vessels between compartments and by providing *asymmetric side-to-center* or *symmetric center-to-side* flow vascular morphogenesis is enhanced in terms of overall network length. The developed open-top microfluidic chip may find various applications in generation of tissue-specific 3D-3D co-cultures for studying cellular interactions in vascularized tissues and organs.

**Supplementary Information:**

The online version contains supplementary material available at 10.1038/s41598-024-74493-3.

## Introduction

Organ-on-chips (OoCs) are microscale devices aiming to mimic the structural and functional characteristics of human organs to better understand their physiological behavior and response to diseases and drugs. OoCs consist of determined compartments for cultured cells, hydrogel(s), and growth medium supplied with perfusion to capture in vivo–like three-dimensional (3D) tissue microenvironment^1,2^. Compared to traditional *in vitro* platforms, OoCs resolve several major challenges of organotypic research: first, the compartment geometry can be adjusted to resemble the in vivo structure of the studied tissue and increase the ‘morphological resolution’ by designing specific sub-compartments for different tissue elements such as a vascular compartment^3^. Second, compartment dimensions closely mimic a physiological ‘fluid to tissue’ ratio and are more comparable to in vivo tissue microenvironment^4^. Third, microfluidic devices allow the generation of gravity-driven or pump-assisted medium flow around vasculature, interstitial flow, replenishing medium from hydrogel-embedded cells to provide active transport of nutrients and remove waste products . In addition, interstitial flow may be utilized as a cue for mechanosensing cells such as endothelial cells (ECs), which are reported to benefit from a physiological shear stress^5–7^. The described solutions demonstrate the potential of microfluidic OoCs as a methodological development in basic research to generate complex yet controllable organotypic cultures.

One of the key features in nearly every human tissue is microvascular network, which is responsible for exchanges of gases, nutrients, metabolites, and a vast range of biochemical molecules in various physiological processes^8^. Vascular network formation occurs via vasculogenesis, where endothelial precursor cells, angioblasts, self-assemble into a vascular plexus giving rise to the forming network de novo, or via angiogenesis, in which endothelial cells form new branches from pre-existing vessels via VEGF-signalling^9^. During vessel formation, vascular-supporting cells are recruited from extravascular space to facilitate lumen formation and vessel stabilization via several signaling pathways, e.g. platelet-derived growth factor signaling pathway (PDGF-BB: PDGRFb) between endothelial cells and vasculature-supporting cells^10,11^. The immediate crosstalk between the forming microvasculature and extravascular cells with the surrounding stroma in vivo highlights the dynamic relationship in microvascular network generation. Another factor affecting vessel formation is the presence of interstitial flow^5^. It has been reported that biochemical cues together with interstitial flow enhance lumen formation and promote EC morphogenesis^5^. Therefore, it is of great importance to include appropriate vasculature-supporting cells with endothelial cells and provide interstitial flow across the cell culture compartment to better recapitulate tissue vascularization *in vitro*.

Over the past decade, an increasing number of OoCs examining self-assembling microvascular network formation and network characteristics have been reported^5,12–21^. These studies focus on characterizing the self-assembled microvascular network as a co-culture of hydrogel-embedded ECs and vasculature-supporting cells. In the context of microfluidic devices, the described methodology has been mostly applied in vascularizing one distinct, closed compartment. Indeed, current device designs consist of a cell culture compartment with two adjacent medium channels restricted mechanically by using micro-posts that control the hydrogel loading with tension^22^. Another alternative for restricting hydrogel into the desired compartment is the utilization of phaseguides, in which the advancing hydrogel aligns itself along with the predetermined geometry^23^. Phaseguides have been used in some microfluidic devices to guide the loading of hydrogel maintaining the hydrogel in its own compartment and preventing the leakage of hydrogel to adjacent medium channels^24,25^. Though the usage of phaseguides increases the dynamic area between the hydrogel-embedded cells and medium, all transport processes are still limited to the lateral direction of diffusion compromising in vivo-like mimicry of transport processes^22^. Alternatively,* in vitro* microvessel study models are generated by using prepatterned microchannels^26,27^. These microchannels are manufactured with varying methods, like casting hydrogel around a sylindrical rod or mold^19,28–30^ or viscous finger patterning^31–33^, which are ultimately coated with ECs. More recently, open microfluidic device designs for integrating two 3D cell culture compartments in immediate contact to investigate multicellular interactions have been introduced. Paek et al. (2019) developed a sophisticated ‘open top’ device for investigating vascular network interplay with adipose stem cells (ASCs), retinal pigment epithelial cells or adenocarcinoma cells to mimic adipose tissue, blood-retinal barrier, or vascularized solid tumor, respectively. The adjacent medium channels allowed a complete dynamic interface between hydrogel-embedded cells and medium as the channels were formed after hydrogel gelation^28^. Atop the hydrogel compartment, a “lower compartment”, Paek et al. constructed an open compartment, an “upper compartment”. They reported 3D vascular network formation in the lower compartment with a monolayer of studied cells introduced in the upper compartment. Similarly, Lin et al. (2021) reported an open top well plate design comprised of fibrin-embedded 3D vascular network with liver spheroids with adjacent medium reservoirs connected via tunnels that allowed bi-directional flow through the culture compartment. In both studies, they verified 3D-2D cellular communication between the compartments, yet 3D-3D communication including a hydrogel-embedded cell culture in the upper compartment was not reported^28,34^.

Another open top device design by Jones et al. (2022) introduced a vascularized skin-on-a-chip model potentially used as a platform to examine the systemic delivery of therapeutics. The study was established into a similar open top device in which they reported the generation of two 3D cultures, including an upper avascular, epidermal compartment and a lower dermal compartment with vascularization^35^. However, the flow across the cell culture compartments was not characterized as the fluid flow was found biologically non-significant in terms of cell maturation. A study by Young et al. (2021) compared multiple designs of single-plane and dual-plane microfluidic devices including both adjacent, perfusable channels and stacked channels to study the multidirectional diffusion affecting hydrogel-embedded cells. Still, the device is applicable to study only one hydrogel compartment which can be considered a limiting factor of the design. Another study by Bonanini et al. (2022) described an open top microfluidic device with bi-directional flow of the adjacent barrier-free medium channels allowing to cultivate a liver spheroid culture on top of the vasculature. Though the reported structure^36^ would allow plating of two 3D cultures, the state-of-the-art studies did not report such experiments.

To our knowledge, we are first to report a microfluidic, open-top device with a barrier-free lower compartment and an open upper compartment to facilitate two 3D culture compartments, in which cells in both compartments are cultured under fluid flow. As a proof-of-concept, we generated vascular networks in the upper and lower compartments to verify connection of the compartments through development of interconnected microvascular networks. We quantified the vascular network volume, the mean diameter, and the overall length of the vasculature, verified distinct characteristics between the flow conditions, and showed in vivo-like density of the formed vasculature. In addition, microvascular networks in all flow conditions showed lumenized vessels with a physiologically relevant diameter. Finally, we demonstrate the feasibility of the developed platform having two distinct, yet barrier-free 3D cell culture compartments allowing complete integration of cells from compartment to compartment.

## Materials and methods

### Fabrication and design of the barrier-free open top chip

**Fig. 1:**
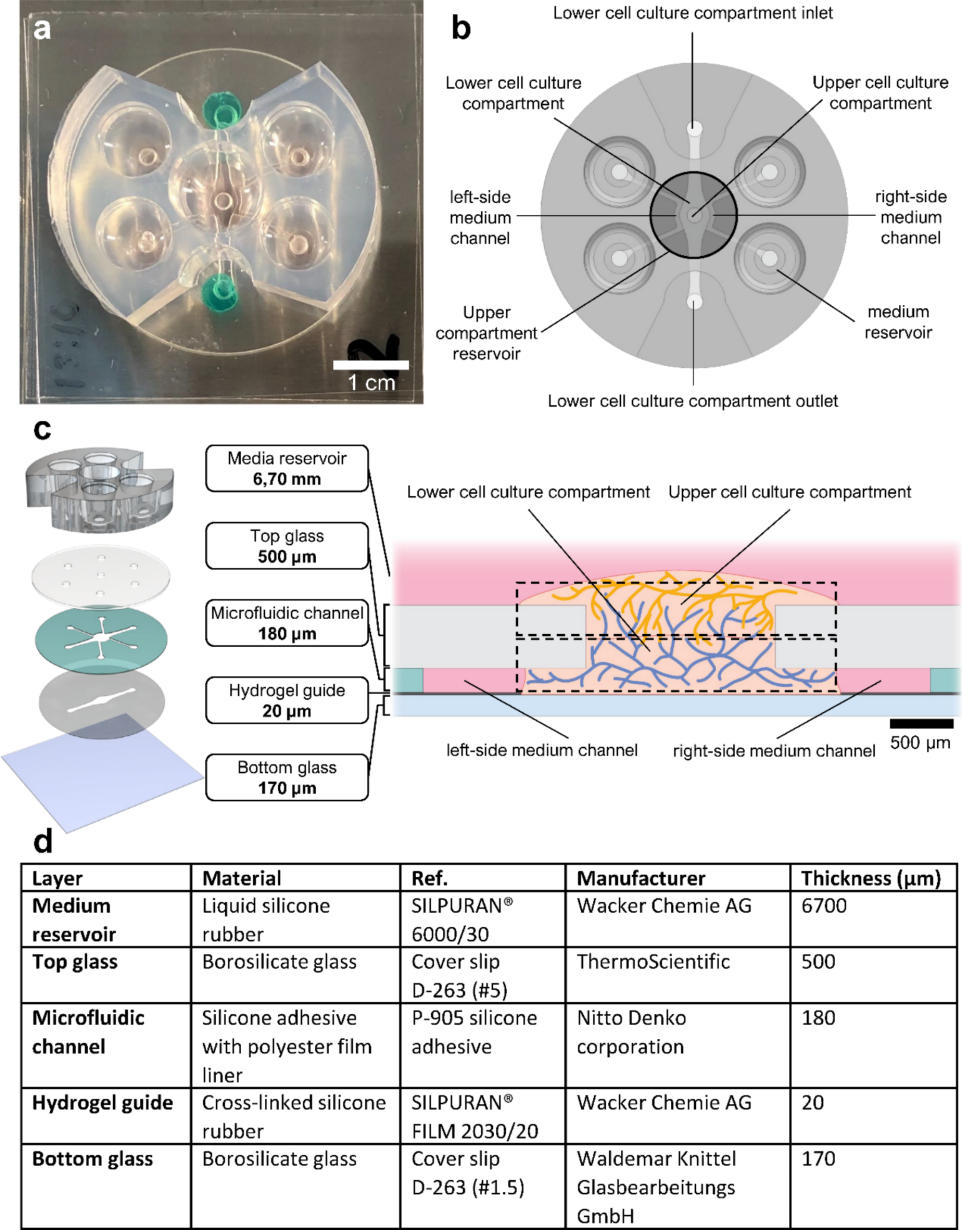
Open top chip for 3D-3D cell culture. **a** Photo of the barrier-free open top chip in culture. Scale bar 1 cm. **b** Schematic representation of the open top chip with compartment naming. Image composed with 3D CAD Design software SolidWorks. **c** Open top chip design utilizes a stacked five-layer architecture founded on borosilicate bottom glass. Hydrogel guide, microfluidic channel and top glass layers are assembled to establish a compartment for lower cell culture compartment (blue vessels) and adjacent medium channels. The medium reservoir layer consisting of five reservoirs allows the addition of media and generation of the chosen gravity-driven flow. The top glass layer includes an opening to be utilized as a compartment for the upper cell culture (yellow vessels) and provides inlets for medium addition to medium channels for the lower compartment. Cell culture compartments, and medium channels are indicated with dashed boxes. Scale bar 500 μm. Images composed with 3D CAD Design software SolidWorks and Adobe Illustrator. **d** Specifications of materials used for the different layers of the chip.

The barrier-free open top chip (Fig. 1A and B) developed in this study is comprised of five layers: (1) a bottom glass layer, (2) a hydrogel guide layer, (3) a microfluidic channel layer, (4) a top glass layer and (5) a medium reservoir layer (Fig. 1C). Specifications of the materials used are described in Fig. 1D. In short, the open top chip is fabricated on top of a cover slip glass, the bottom glass layer. The hydrogel guide layer restricts the hydrogel from leaking to the adjacent medium channels, which are formed with the microfluidic channel layer and the top glass layer. The medium channels in the microfluidic channel layer connect the medium reservoirs and the cell culture compartments and allow the generation of gravity-driven, interstitial fluid flow across the two cell culture compartments. The upper cell culture compartment is centered atop the lower cell culture compartment and is equipped with an independent upper compartment reservoir (Fig. 1B, darkened circle). The lower and upper cell culture compartments are depicted in Fig. 1C.

The chip and channel structures were fabricated from separate layers onto a borosilicate cover slip glass (#1.5, 38 mm x 38 mm, 170 μm, Waldemar Knittel Glasbearbeitungs GmbH Braunschweig, Germany) suitable for high-resolution confocal imaging. The layers were designed with SolidWorks (Dassault Systèmes, Vélizy-Villacoublay, France) and designs were exported to DXF files for processing with laser cutters. The microfluidic features of the hydrogel guide layer and the microfluidic channel layer were fabricated by laser cutting with a Speedy 100 CO_2_ laser system (Trotec, Marchtrenk, Austria). The hydrogel guide layer was cut from a 20 μm thick Silpuran^®^ film (Wacker, München, Germany) with 6.6 W power, 14 mm/s speed, and 10 kHz frequency. The microfluidic channel layer was cut from a stack of two 90 μm thick layers of a double coated polyester silicone adhesive tape (P-905, Nitto, Osaka, Japan) using the laser with 8.7 W power, 17 mm/s speed, and 3 kHz frequency. Inlets and co-culture opening were cut into a 500 μm thick circular (ø 35 mm) borosilicate glass (Thermo Scientific, Waltham, MA, USA) with a femtosecond laser system. The femtosecond laser system consisted of a laser (Pharos 20 W, Light Conversion, Vilnius, Lithuania), an attenuator (LPA, Optogama, Vilnius, Lithuania), an expander (MEX, Optogama, Vilnius, Lithuania), a scanner (hurrySCAN II 14, Scanlab, München, Germany), and a movement device (H-840.D21, Physik Instrumente, Karlsruhe, Germany). The parameters used were 22 µJ pulse energy, 343 nm wavelength, 175 mm/s speed, 25 kHz frequency with a 4 μm beam width. The hydrogel guide and microfluidic channel layers were examined to be cytocompatible (Fig. S1).

The fabricated layers were cleaned thoroughly before assembling. The laser fabricated borosilicate glasses were pre-cleaned in detergent solution and tap water. All the glass parts were cleaned by submerging them sequentially in acetone and isopropanol in an ultrasonic cleaner. Afterwards, they were rinsed with deionized water and dried with filtered (0.01 μm) compressed air. The laser-cut hydrogel guide layers were cleaned by submerging them first in isopropanol and then in deionized water and were finally left to air dry. The surfaces of the laser-cut microfluidic channel layers were wiped clean with deionized water leaving them to air dry.

Injection-molded Silpuran^®^6000/30 part (Wacker, München, Germany), named as 6-well, was used to construct the medium reservoir layer^37^. Each 6-well device was modified before disinfection and bonding. The upper compartment reservoir was cut using a circular biopsy punch (ø = 8 mm) and the reservoirs of the original 6-well device located at the places of the lower compartment inlet and outlet were detached using a circular biopsy punch (ø = 6 mm) and a scalpel.

Next, all medium reservoirs were made hydrophilic using a similar method reported previously^38^. In the present study, Mw ~ 29,000 polyvinylpyrrolidone (PVP) (Sigma-Aldrich, 234257-100G, 18.17% (w/w)) was used. In brief, the surface of the medium reservoir layer was activated with oxygen plasma using a low-pressure plasma system with 30 W power, 60 s duration and 30 mbar pressure (Diener electronic GmbH + Co. KG, Ebhausen, Germany). Then, the medium reservoir layer was incubated in the PVP solution for 10 min and rinsed with deionized water.

The assembly of the five layers into the finished chip was conducted in four steps. First, the hydrogel guide layer was bonded onto the bottom glass layer with oxygen plasma (30 W, 15 s, 30 mbar). Second, the microfluidic channel layer was manually aligned and pressed onto the top glass under a digital microscope. Third, the top glass layer attached with the microfluidic channel layer was manually aligned and pressed under the digital microscope onto the bottom glass layer attached with the hydrogel guide layer. Subsequently, the microfluidic chips and the medium reservoir layers were disinfected by submerging them in 70% ethanol. Residual ethanol was aspirated from the chips, and the medium reservoir layers were air dried after the treatment. After full drying, medium reservoir layers and temporary pipetting guides were manually aligned and bonded reversibly on the chips.

### Contact angle measurements

Contact angle measurements were conducted with the sessile drop method using Attention Theta Lite Optical Tensiometer (Biolin Scientific, Gothenburg, Sweden). Static contact angle values were measured from static droplets (volume = 5 µl) with OneAttension software (Version 3.2, Biolin Scientific). The mean contact angle results (*n* = 3) were calculated from the means of the recorded contact angle values of different materials with duration of 10 s and 10 fps frame rate from both edges of the droplets.

### Flow simulation and analysis

To analyze different flow conditions used in this study, three different flow conditions were studied using COMSOL Multiphysics (Version 6.1 COMSOL, Inc., Burlington, USA). Free and Porous Medium Flow was applied for modelling the flow across the hydrogel compartments. For the liquid phases, the incompressible flow formulation of the Navier-Stokes equation was used, whereas in hydrogel, which was modeled as porous media, the slower flow was governed by the Brinkman equations. The following material parameters were used in the simulations: density 993,3 kg/m^3^, dynamic viscosity 0,6913 Pa s, and porosity and permeability of hydrogel 0,99 and 6,67 × 10^−12^ m^2^, respectively^15,20^. Excess hydrogel above the top glass originated from pipetting the upper cell culture compartment (Fig. 1C) was modeled as a spherical cap with a 49° contact angle based on the contact angle measurement. Furthermore, it was estimated that the height and the radius of the base of the cap were 1,1 mm and 2,2 mm, respectively, resulting in a volume of the spherical cap as approximately 8 µl.

### Optimization and demonstration of the gravity-based flow

Multiple approaches for producing differing flow profiles (Fig. S1) were applied to study the effect of hydrostatic pressure and flow on microvascular network formation. In Approach 1, 200 µl and 50 µl of medium was pipetted to left-side and right-side reservoirs, respectively, to produce medium flow occurring towards right-side reservoirs. To balance the hydrostatic pressure of the upper compartment reservoir with the right-side reservoirs, 115 µl of medium was applied to the upper compartment reservoir. In Approach 2, minimal flow was applied by using the end volumes of Approach 1, i.e. 150 µl of medium was applied to the upper compartment reservoir and 116 µl to the left- and right-side medium reservoirs. In Approach 3, 200 µl of medium was applied to the upper compartment reservoir and 50 µl to the left- and right-side medium reservoirs to maintain flow from the upper compartment to the lower compartment. In Approach 4, 330 µl of medium was applied to the upper compartment reservoir, and 50 µl to left- and right-side medium reservoirs to maximize both hydrostatic pressure and flow duration in the cell culture compartments. In Approach 5, all reservoirs were fully filled, i.e. 200 µl in the medium reservoirs and 330 µl in the upper compartment reservoir to minimize flow and maximize hydrostatic pressure.

Three flow conditions, Approach 1–3, were selected and are further referred as *asymmetric side-to-center* (Approach 1), *symmetric side-to-center* (Approach 2) and *symmetric center-to-side* (Approach 3) hereafter. With two of the flow profiles, symmetric flow conditions were produced between the left-side and right-side medium reservoirs and the upper compartment reservoir located in the center of the chip. In the *symmetric center-to-side* flow, the flow occurred from the upper compartment reservoir to the adjacent reservoirs, while in the *symmetric side-to-center* flow profile, the flow direction was reversed. To compare the *symmetric side-to-center* flow condition with a flow condition where the flow direction is from solely one side to the center, we applied an *asymmetric side-to-center* flow profile.

To visualize the flow in *asymmetric side-to-center* condition, 100 µg/ml solution of 70 kDa Rhodamine B isothiocyanate–Dextran (R9379, Sigma) in DPBS was flushed into the medium reservoirs to record moving flow front within the acellular, fibrin-laden culture compartments (Fig. S1B and Supplementary video 1). For *symmetric center-to-side* condition, acellular, fibrin-laden chips were incubated with the same fluorescent solution and recorded the flushed-out flow front (Fig. S1C and Supplementary video 2). Sequential images were acquired to observe the flow characteristics.

### Human cell culture

Human ASCs were isolated from subcutaneous abdominal tissue samples obtained from one donor. Tissue samples were obtained at the Tampere University Hospital Department of Plastic Surgery with the donor’s written informed consent and processed under ethical approval of the Ethics Committee of the Expert Responsibility area of Tampere University Hospital (R15161). The cells were isolated as described previously^39^. The ASCs were cultured in α-MEM supplemented with 5% human serum (HS) (Serana Europe, Germany), 100 U/ml penicillin, and 100 µg/ml streptomycin and used in passages 3–5. The mesenchymal origin of ASCs was previously confirmed by surface marker expression analysis with flow cytometry and assessment of adipogenic and osteogenic differentiation potential^20^.

Human Umbilical Vein Endothelial Cells (HUVECs) expressing green fluorescent protein (GFP-HUVECs) and red fluorescent protein (RFP-HUVECs) were commercially obtained from Cellworks (Caltag Medsystems Company, Buckingham, UK) and Angio-Proteomie (Boston, USA), respectively. Both fluorescently labeled HUVECs were cultured in Endothelial Cell Growth Medium-2 Bullet Kit (EGM-2; Lonza, Switzerland) consisting of Endothelial Cell Growth Basal Medium (EBM-2) and Endothelial Cell Growth Medium-2 Supplements. Instead of the fetal bovine serum supplied with the Kit, 2% HS (Serana Europe, Germany) was used. The cells were used between passages 3 and 6.

Two microvascular networks were generated on top of each other in the lower and upper cell culture compartments. For generating the lower compartment vascular network, GFP-HUVECs with ASCs were used to generate ASC-supported GFP-HUVEC vascular network, referred as GFP-vasculature hereafter. Similarly for the upper compartment vasculature, RFP-HUVECs and ASCs were used to generate ASC-supported RFP-HUVEC vascular network, referred as RFP-vasculature hereafter. Microvascular networks were formed through self-assembly of HUVECs and ASCs in fibrin hydrogel according to our previous work^20^ with the following modifications. Briefly, two cell suspensions were made: ASCs were combined with either GFP-HUVECs or RFP-HUVECs at 1:5 cell ratio (final cell density 6 million cells/ml) and spun down. The cell pellets were then resuspended in 2 IU/ml human thrombin (Sigma-Aldrich) in EBM-2 and combined at 1:1 volume ratio with 5 mg/ml human fibrinogen (Sigma-Aldrich) in Dulbecco’s Phosphate Buffered Saline (DPBS; Lonza). The resulting mixture (15 µl) was quickly pipetted into the lower cell culture compartment from its inlet to its outlet, which were subsequently closed for restricting evaporation of the hydrogel-cell suspension and stabilizing the inner microenvironment in terms of pressure. The cell-laden fibrin gel was allowed to polymerize in a humidified chamber for 15–30 min in a cell culture incubator (+ 37 °C, relative humidity 95%, 5% CO_2_).

Similarly, the upper compartment vasculature was prepared as described above. The resulting 1:1 mixture of 10 µl fibrinogen-cell suspension was quickly pipetted on top of the lower cell culture compartment into the upper cell culture compartment and let to polymerize in a humidified chamber for 10 min in a cell culture incubator. Due to complete filling of upper cell culture compartment, the excess cell-laden fibrin gel formed a dome-like geometry onto top glass (Fig. 1C). Microvascular networks were cultured in EGM-2 with 2% HS (Serana Europe, Germany). First, medium channels were filled. Next, the three selected flow conditions, *asymmetric side-to-center*, *symmetric center-to-side* and *symmetric side-to-center*, were applied (Fig. 3) to study the effect of flow on the GFP-RFP vascular network formation. Medium changes were repeated daily and the described volumes generating the flow were kept constant throughout the 5-day culture (D0-D4).

To study the overall network formation, daily images of GFP-and RFP-vasculatures were captured with a widefield fluorescence inverted microscope (Leica DMi8) as 3 × 3 tile scans using HC FL PLAN 10x/0.25 air objective. The height of the GFP-RFP-vascular network was measured as a ‘GFP-to-RFP’ principle, i.e. lowest GFP-tagged HUVEC to highest visible RFP-tagged HUVEC to examine hydrogel integrity in z-direction imaging from bottom-to-top. The interface between the cell culture compartments was imaged as a z-stack using N PLAN L x20/0.35 air objective to capture both GFP-and RFP-vasculatures.

### Cytochemical staining

The cytochemical staining protocol was performed by using the *asymmetric side-to-center* flow condition (Fig. S1). After five days of culture, microvascular networks were washed with DPBS and fixed with 4% paraformaldehyde (15713-S, EMS, Hatfield, U.S.) in DPBS for 30 min at RT following by three washes with DPBS. The samples were then permeabilized with 0.1% Triton X-100 (Sigma-Aldrich) for 10 min and non-specific binding was blocked with blocking buffer (1% bovine serum albumin (BSA; Sigma-Aldrich), 0.1% Triton X-100 in DPBS) for two hours at RT. Phalloidin (AD643-81, 1:500, ATTO-TEC, Siegen, Germany) was used to identify the presence of F-actin cytoskeleton, and DAPI, to visualize cell nuclei. Phalloidin and DAPI were diluted 1:500 and 0.67 µg/ml, respectively, in DPBS with 1% BSA and incubated two days at 4 °C. The samples were washed three times with DPBS prior imaging.

### Imaging of the vascular networks

To quantify the 2D vessel distribution in the lower cell culture compartment, tile scan images of the overall GFP-vasculature were captured by using Leica DMi 8 widefield fluorescence microscope x10 dry objective. To assess the 3D morphology of GFP- and RFP-vasculatures in both cell culture compartments, open top chips were imaged with Nikon A1R + laser scanning confocal microscope using Nikon Plan Apo VC 20x DIC N2 objective (air, NA 0,75), NIS-Elements AR software version 5.11.00. Multiple regions of interest (ROIs) were captured from each flow condition (n_asymmetric side−to−center_=10, n_symmetric center−to−side_=10, n_symmetric side−to−center_=9) with a fixed frame size of 2048*2048 pixels with varying height. The ideal sampling rate was calculated with the Nyquist calculator by Huygens SVI (Scientific Volume Imaging, The Netherlands) to avoid undersampling, resulting in a voxel size of 0,13 × 0,13 × 0,576 µm^3^. ROIs were acquired randomly from the stacked cell culture compartments, with predetermined approximations for the xy-coordinates for an appropriate representation of both upper and lower cell culture compartments.

### Quantification of the vascular networks

A quantitative analysis of the 2D GFP-vasculature was performed with Fiji ImageJ. Captured images (10x, 16-byte) were converted into 8-byte, adjusted in terms of contrast (enhance contrast 0.35%) and binarized by using LabKit^40^ to quantify the detected signal of the formed vessels. Binarized percentual area of the vasculature was measured with ImageJ from each ROI (n_asymmetric side−to−center_=5, n_symmetric center−to−side_=3, n_symmetric side−to−center_=4). Unpaired student’s T-tests were used for significance testing between the measured percentage of binarized pixels of the left- and right-side vasculatures.

A quantitative analysis of the 3D GFP-vasculature was done using Imaris Interactive Microscopy Image Analysis software, version 10.0.0 (Bitplane, Belfast, United Kingdom). To prepare the image stacks to Imaris, image stack files were deconvoluted using Huygens Essential version 22.10 (Scientific Volume Imaging, The Netherlands). To compensate for the z-dimensional decrease in fluorescence signal intensity, layer normalization was applied to all channels. Linear Stretch method was applied for the GFP-channel to set the contrast on a range from 0 to 3000 for comparative analysis between the ROIs (Supplementary text file S1 and S2). Quantitative values of the GFP-vascular network volume, the vessel diameter and the total network length per each flow condition were generated with Imaris. Specifically, the vascular volume was computed from a Surface-object of the GFP-channel. The total vessel length and the mean vessel diameter were calculated from created Filament-object based on the GFP-channel masked with the GFP-surface. The absolute vascular volume was divided by the total volume of the captured ROI resulting in a vascular volume percentage of the ROI considering the varying height of the captured ROIs. The mean vessel diameter was computed from the filament occupying the whole ROI. The total vessel length was divided by the total volume of the ROI allowing comparisons between original ROIs. Due to suboptimal fluorescence signal of RFP-HUVECs, quantification was not applicable for the RFP-vasculature.

Median values were used to describe vascular network parameters. The decision to use the median values resulted from the observation that in most cases, we did not observe Gaussian distributions for the vascular volume, the vessel diameter, and the vessel length within raw values in each ROI. Thus, descriptive statistics were reported as violin plots with medium smoothing showing medians with respective percentiles as these are unaffected by non-symmetric distributions. A Spearman correlation was performed to test if there was a relationship between the vascular volume, the vessel diameter, and the vessel length within the flow conditions.

All statistical analyses were computed with GraphPad Prism (version 9.0.0).

## Results

### Characterization of the hydrogel guide showed appropriate contact angles for maintaining hydrogel in lower cell culture compartment

**Fig. 2:**
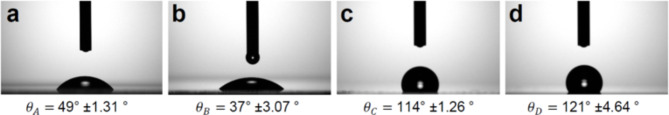
Illustration of wettability of chip materials. **a** Measured water contact angles are presented for top glass untreated borosilicate glass, **b** bottom glass plasma-treated borosilicate glass after 21 days and for **c** untreated Silpuran film and d untreated polyester silicone adhesive tape. All presented values represent means of recorded contact angle values with standard deviations (*n* = 3).

To achieve a barrier-free contact between the cell culture medium and cell-laden hydrogel, a hydrogel guide was introduced within the chip. In addition to a physical restriction, the hydrogel guide relies on different wettability properties of materials to restrict the hydrogel from flowing to the adjacent medium channels^23^. To keep the hydrogel in the lower cell culture compartment, the hydrogel guide material should be hydrophobic while the top and bottom glass layers benefit from hydrophilicity. Therefore, we measured the contact angles of the chip materials (Fig. 2). The mean static contact angles for the untreated borosilicate glass for the top glass layer, silicone rubber film for the hydrogel guide layer and silicone adhesive film for the microfluidic channel layer were 49° ±1.31°,114° ±1.26° and 121° ±4.64° (*n* = 3), respectively. The surface of the hydrogel guide was covered during the plasma treatment to maintain its hydrophobic characteristics. The bottom glass layer is exposed to plasma treatment that decreases the measured static contact angle down to < 2°. However, as cells were not seeded directly after the chip assembly, we measured that over the course of 21 days, the contact angle increased only up to 37° ±3.07° maintaining the hydrophilic characteristics. The microfluidic channel layer does not take part in hydrogel guiding but forms the walls for the microfluidic channels mediating the gravity-driven flow.

### Applied flow conditions maintained hydrogel integrity and enabled vessel formation

We conducted multiple approaches (Fig. S2) to produce flow across the lower and upper cell culture compartments. To assess the results, we evaluated the cell-laden hydrogel integrity in terms of decrease in GFP-to-RFP signal representing the height of the combined microvascular networks in the z-axis direction, referred as GFP-to-RFP-height hereafter, during the culture period. We determined the hydrogel integrity to be acceptable with maximum of 15% decrease in height at D4 compared to D0. In the *asymmetric side-to-center* flow, we observed well-established 3D vasculatures and the measured GFP-to-RFP-height decreased maximum of 9% at D4 compared to D0 (Fig. S3). In symmetric side-to-center flow condition the reduction was 11% at D4 compared to D0 and in the symmetric center-to-side flow condition, the maximum decrease was 14% at D4 compared to D0. Conversely, in Approaches 4 and 5, the maximal supply of medium either to the upper compartment reservoir or all the reservoirs, we observed formed microvascular networks with greatly decreased GFP-to-RFP-height (data not shown). Based on accepted percentual decrease in GFP-to-RFP height, we continued with the confirmed three flow conditions for further experimental work.

### Simulated flow revealed distinct differences in flow patterns

**Fig. 3:**
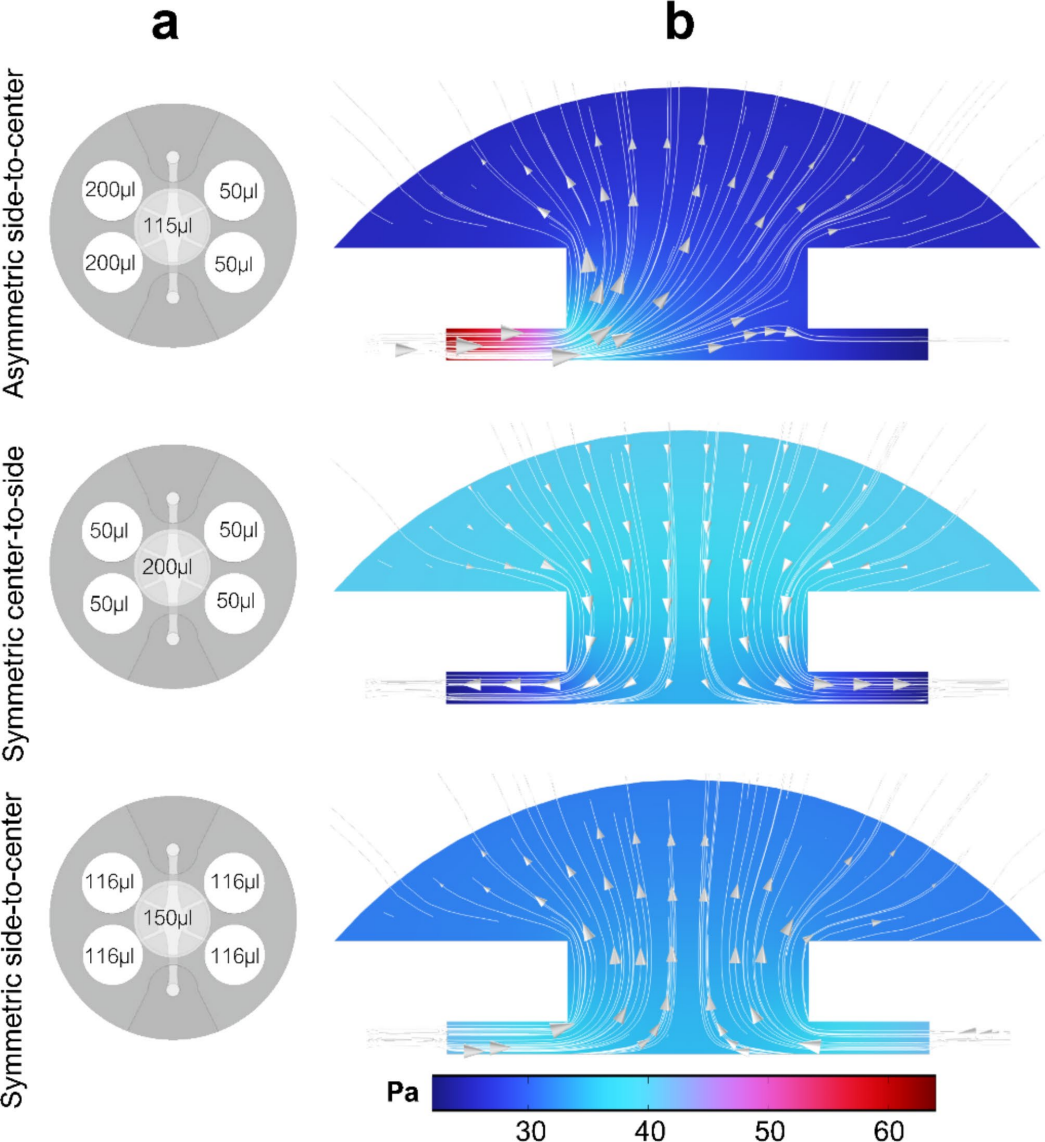
Generation of studied flow profiles asymmetric side-to-center, symmetric center-to-side and symmetric side-to-center. **a** Used volumes were introduced every 24 h to refresh medium and recreate fluid flow across the culture compartments. **b** Simulated pressure (Pa) depicts the pressure gradient of initial volumes in each flow condition (streamline: velocity field).

We performed numerical simulations to examine initial pressure distributions inside the lower and upper cell culture compartments in all the selected three flow conditions. Medium volumes applied into the different reservoirs (Fig. 3A) generated initial pressure differences, resulting transient, gravity-driven flows across the cell culture compartments. Using the initial medium volumes, we simulated the 2D profiles of the pressure distributions inside the hydrogel (Fig. 3B). Simulated pressure in the *asymmetric side-to-center* condition shows that pressure-induced flow is asymmetrically divided between the lower and upper cell compartments, compared to other two cases where the flow is symmetric. Furthermore, the pressure difference is largest in the *asymmetric side-to-center* condition, where approximately 40 Pa initial pressure difference is created compared to pressure differences of 18 Pa and 10 Pa for the *symmetric center-to-side* and *symmetric side-to-center* conditions, respectively. This results in the highest initial pressure-induced flow rate in the *asymmetric side-to-center* condition.

To support our simulation data on flow occurring inside the lower and upper cell culture compartments, we visualized the moving flow front in *asymmetric side-to-center* and *symmetric center-to-side* conditions (Fig. S2 using fluorescent 70 kDa dextran molecules in the flowing fluid. In Approach 1, we observed the moving flow front penetrating the lower cell culture compartment and continuing to rise to the upper cell culture compartment (Supplementary video 1). In Approach 5, we observed decreasing intensity of dextran in the upper cell culture compartment and indicating the dextran molecules flushing away from the upper to lower cell culture compartment (Supplementary video 2).

### Vascular network formation occurred in all flow conditions

**Fig. 4:**
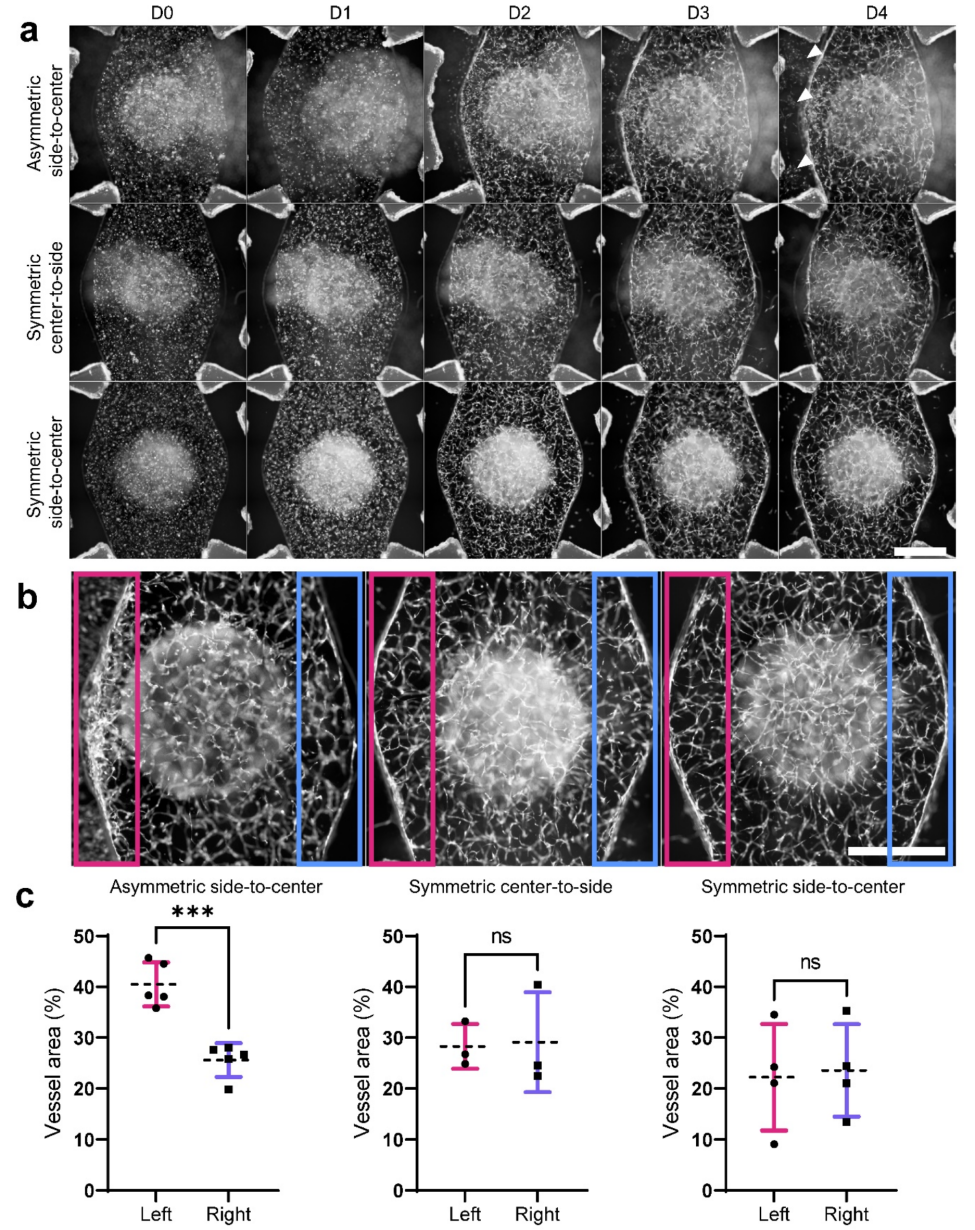
Overall development of GFP-vasculature. **a** Daily development of GFP-HUVEC vascular network on open top chip under different flow conditions ‘asymmetric side-to-center’, ‘symmetric side-to-center‘ and ‘symmetric center-to-side’. Scale bar 1 mm. **b** Observed morphological differences in GFP-vasculature in asymmetric side-to-center condition. A denser area of clustered GFP-HUVECs formed against flow (pink box) in asymmetric side-to-center condition compared to adjacent side (blue box). In symmetric flows, clustered GFP-HUVECs were not observed. Scale bar 1 mm. **c** Comparison of the quantified vessel area between left (pink box) and right (blue box) sides in lower cell culture compartment. Scatter plots demonstrate the individual values with means (dashed line) and standard deviations (whiskers). *** denotes p < 0.001, ns denotes non-significant with Unpaired student’s t-test.

To examine microvascular network formation in both cell culture compartments in tested flow conditions, we demonstrated the formation of branched, ASC-supported GFP-RFP-vasculature within five days of culture (Fig. 4A). At D4, GFP-vasculature showed differences in the overall morphology and the network assembly around the open top area. In the *asymmetric side-to-center* flow, GFP-HUVECs were observed to migrate towards the left medium channel against the direction of left-right flow. In the *symmetric center-to-side* and symmetric side-to-center conditions, we observed a few GFP-HUVECs migrating towards both medium channels, still, less extensively compared to the *asymmetric side-to-center* flow. In addition, we observed a denser area of clustering GFP-HUVECs as a robust monolayer-like assembly forming to the left side of the *asymmetric side-to-center* condition compared to the other conditions (Fig. 4B). Using on image quantification, we showed a significant difference of vessel area between the sides.

Similarly, we detected RFP-vasculature formation in the upper cell culture compartment (Fig. S4). However, due to optical limitations of widefield microscopy, further observations of RFP-vasculature were performed with confocal microscopy.

### Applied flow conditions resulted in interconnected vasculatures with open lumens

**Fig. 5:**
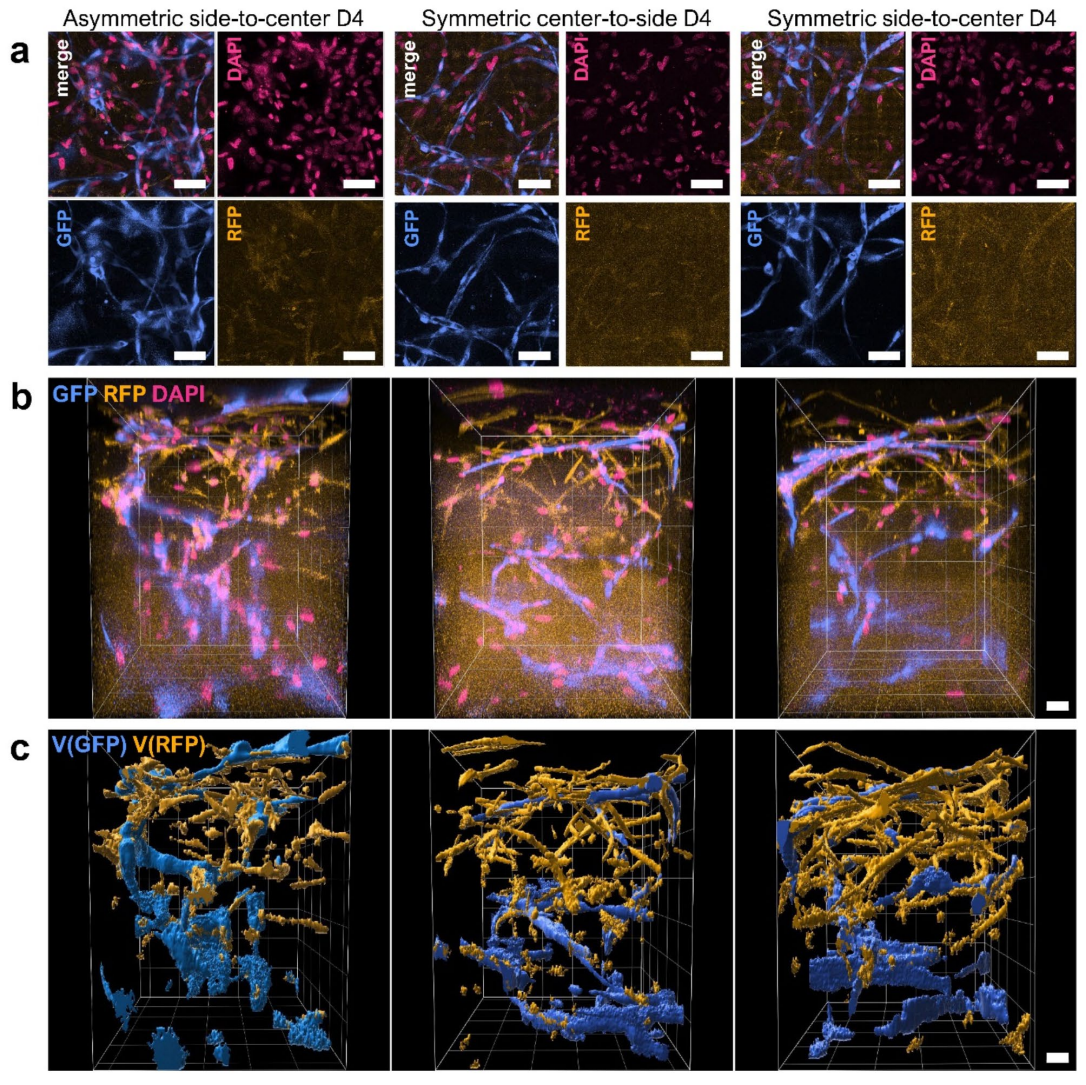
Overall development of GFP-RFP-vasculatures. **a** GFP-RFP-vasculature in open top area shown as Maximum Intensity Projections (MIPs). ASC-supported vascular networks of GFP-HUVECs (blue) and RFP-HUVECs (yellow) show overlapping signal in all flow conditions. Nuclei stained with DAPI (pink). Scale bars 50 μm. **b** 3D projection of GFP-RFP-vasculature depicting the vascular network distribution in z-direction. GFP-vasculature (blue) and RFP-vasculature (yellow) form merging vessels in all conditions. Scale bar 50 μm. **c** Signal-based surface of GFP-vasculature (blue) and RFP-vasculature (yellow) highlight the vessel distribution and merging structures. Scale bar 30 μm.

**Fig. 6:**
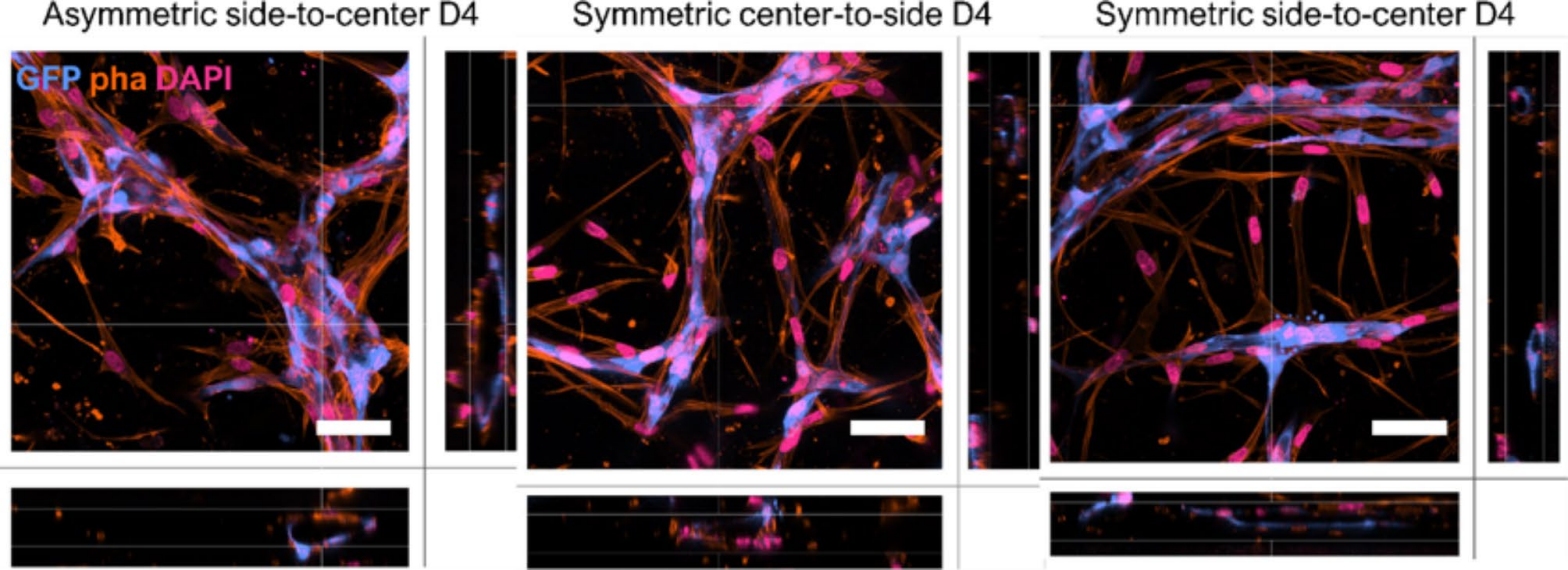
All flow conditions showed lumenized vessels with in vivo-relevant diameter. Orthogonal view depicts the x- and y- projections of the hollow vessel showing continuous endothelial cell lining (GFP, cyan), actin skeletons of GFP-HUVECs and ASCs (phalloidin, orange) and nuclei (DAPI, magenta). Scale bars 50 μm.

Advanced image processing on the captured z-stacks from the open top area revealed the formation of a branched network of RFP-HUVECs on top of the GFP-vasculature (Fig. 5). Maximum intensity projection showed GFP-vessels closely aligning with RFP-vessels (Fig. 5A). Furthermore, 3D projections of the captured z-stacks from the open top region further verified not only the presence of interconnected, branched GFP-vasculature throughout the lower compartment but also robust branches towards the upper compartment (Fig. 5B). Similarly, the ASC-supported RFP-HUVEC network showed robust branches penetrating to the lower compartment (Fig. 5B). Masked surfaces of GFP- and RFP-signals demonstrated an observable difference between GFP- and RFP-vasculatures showing a larger diameter and overall greater vessel structures with less branched appearance for the GFP-vasculature compared to the RFP-vasculature (Fig. 5B-C). We also verified the presence of vessel lumens in all flow conditions (Fig. 6). Similar to our previous findings^20^, ASCs were shown to closely align with the GFP-vasculature (Fig. S5) demonstrating their presence as supportive, perivascular cells (Fig. 6).

### Descriptive statistics confirmed differences in vascular network parameters between flow conditions

**Fig. 7:**
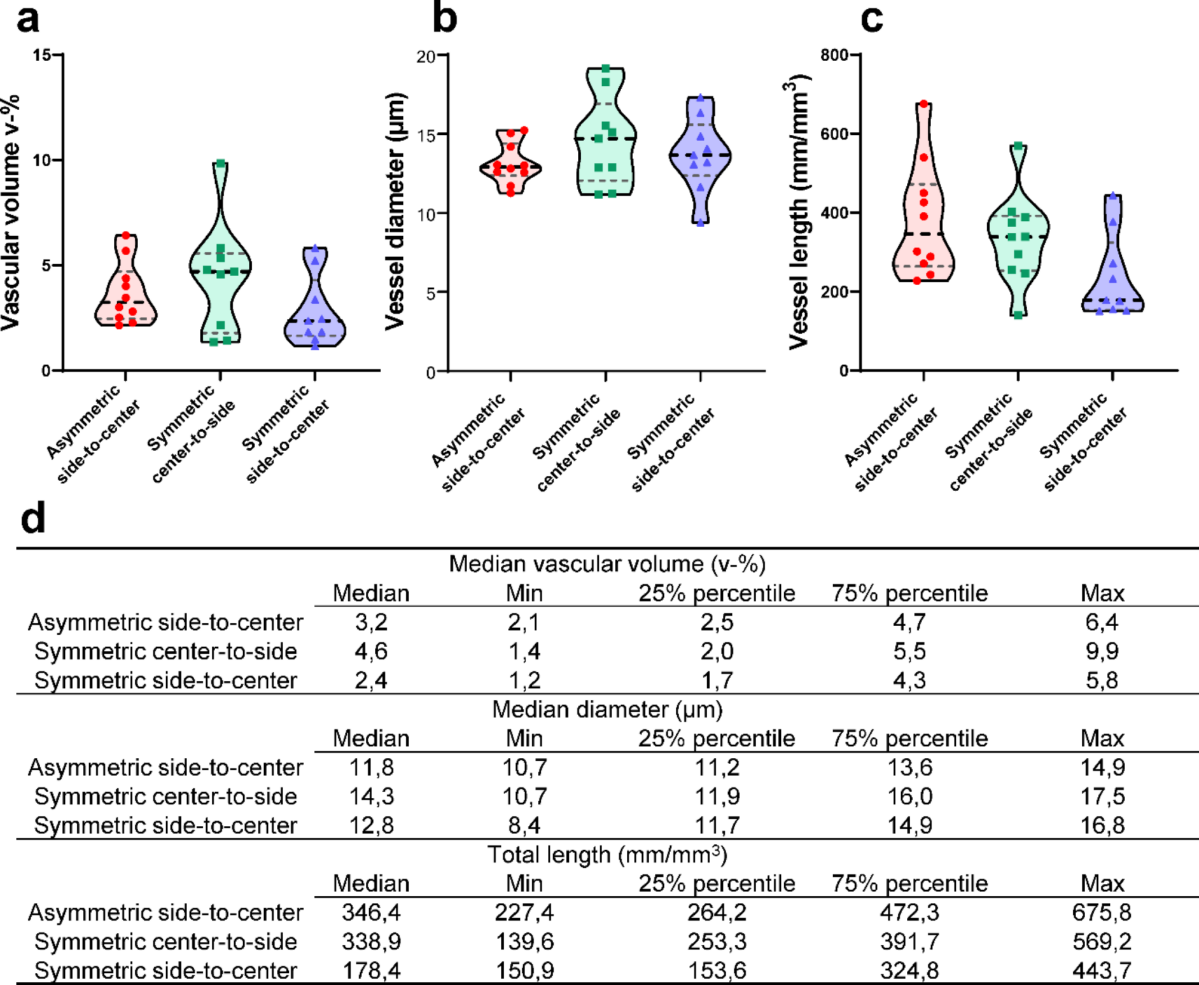
Quantification of vasculature properties. **a-c** Violin plots of vascular network parameters in asymmetric side-to-center (red, *n* = 10), symmetric center-to-side (green, *n* = 9) and symmetric side-to-center (blue, *n* = 9) with individual plotted values. **a** Percentual vascular network volume V-%. **b** vessel diameter (µm). **c** Total network length (µm). **d** Descriptive statistics of the studied parameters.

The RFP-signal failed to provide reliable surfaces and filaments of the RFP-vasculature due to the inadequate nature of the endogenous fluorescent signal. Therefore, the quantitative investigation was solely performed based on the created surfaces and the filaments of the GFP-signal (Table S1). Descriptive statistics of the flow-affected vasculatures revealed both similarities and minor differences between the studied flow conditions (Fig. 7). The median vascular volume is the largest in the *symmetric center-to-side* and *asymmetric side-to-center* conditions compared to the *symmetric side-to-center* condition (Fig. 7A) though it does not reach statistical significance. Yet larger vascular volume attributes to the greater vessel length in the *symmetric center-to-side* and *asymmetric side-to-center* conditions (Fig. 7C). The median vessel diameter resulted in relatively similar values between all the studied flow conditions (Fig. 7B). The *symmetric side-to-center* condition having the lowest initial hydrostatic pressure resulted in the lowest network volume and the smallest total network length. This is in line with our visual observation of the microvasculature in the *symmetric side-to-center* condition to have the narrowest diameter and the least vascular volume (Fig. S6).

Spearman correlation was used to study the relationship of the vascular volume and the vessel diameter and the total length (Table S2). The results indicated that there is a positive association between the vascular volume and the vessel diameter as well as the vascular volume and the total length in all flow conditions. Moreover, we found a significant positive association between the vascular volume and the vessel diameter in the *asymmetric side-to-center* and *symmetric center-to-side* conditions. Similarly, the vascular volume and the total length had a significant positive association in the *symmetric center-to-side* and *symmetric side-to-center* conditions. Within the *symmetric center-to-side* condition, the vessel diameter and the total length had a significant positive association.

## Discussion

Developing open, multi-compartmentalized microfluidic devices is essential for mimicking vascularized tissues and organs *in vitro. *Indeed, recent studies have reported a vascular network together with an implemented monolayer^28^or spheroids^34,36^ of another cell type to examine the physiology of vascularized tissues in the human body. Despite the significant progress in establishing vascularized 3D co-cultures in microfluidic platforms, to our knowledge, there are only few reported studies^35^ with such double 3D cell culture set up. Jones et al. (2022) demonstrated 3D-3D cell culture of avascular epidermal layer together with vascularized dermal compartment. Though the generated skin construct showed physiological relevance in overall morphology, the experimental work was carried out as a static culture^35^. Interestingly, the effect of flow was briefly studied yet it was shown to have no significance on cell maturation^35^. Conversely, medium flow across a vascular network compartment is known to promote vascular morphogenesis together with soluble angiogenic cues^5^ indicating that flow during vascular network development is necessary for better physiological mimicry.

In this study, we developed a barrier-free, open top microfluidic device and validated the design by generating de novo vascular network formation in two overlying culture compartments by using fibrin-embedded fluorescent-tagged HUVECs and ASCs as reported previously^20^. ASCs are known to support the endothelial cell self-assembly acting as pericytic cells and facilitating the forming vasculature^18–20^ making these cells an attractive choice for vascular network generation *in vitro*. Here, the reported 3D vasculatures formed in the two compartments demonstrates the device feasibility to host distinct 3D cell cultures in immediate contact with each other. The developed platform is suitable for introducing gravity-driven interstitial flow to enhance the delivery of nutrients and oxygen through the culture compartments and to promote vascular network formation. To our knowledge, our study is the first to determine the effect of unidirectional, transient flow across the barrier-free cell culture compartments in vascular network formation within an open top microfluidic chip.

Devices with physical restricting structures, such as micro-posts or membranes, can occupy a considerable area between distinct cell culture compartments and between the medium channel and the cell culture compartment. Specifically, the diffusion limit of oxygen and nutrients has been widely recognized to be around 200 μm, also ensuring sufficient metabolic waste removal^18,41^. To further enlarge the biological interface between hydrogel-embedded cells and cell culture medium, some of the microfluidic platforms utilize physical restricting structures e.g. needles, which will provide circular channels for both sides of the cell culture compartment^19,28^. Alternatively, we developed a barrier-free device by introducing two 3D cell culture compartments in direct contact with one another allowing cellular interactions and migration between the sequentially pipetted cell-laden hydrogels. We used hydrophobic surface properties of silicone rubber as a phaseguide to retain hydrogel-embedded cells in the lower cell culture compartment while allowing unrestricted medium supply through the adjacent medium channels. The different contact angles of the materials used - borosilicate glass, silicone rubber film and silicone-polyester adhesive tape - were successfully shown to enable manual hydrogel loading in the lower cell culture compartment simultaneously preventing the hydrogel leakage to adjacent medium channels.

In terms of vascular network formation, interstitial flow is known to promote vascular morphogenesis^5,7,42^. To implement suitable medium flow across the two culture compartments, we simulated different gravity-driven flow conditions to characterize pressure differences indicating the direction of the flowing medium. We showed that the *asymmetric side-to-center* flow produced the largest pressure difference inside the cell culture compartments with the least uniform flow profile. Conversely, *symmetric center-to-side* flow condition was simulated to provide a uniform medium flow across both cell culture compartments. Furthermore, both *asymmetric side-to-center* and *symmetric center-to-side* flow conditions were experimentally shown to have flow fronts similar to the simulated flow conditions verifying the flow behavior in these conditions. In the *symmetric side-to-center* flow condition, the simulated flow was similar to *symmetric center-to-side* flow but in opposite direction. We, however, acknowledge that the complexity of the microfluidic chip with open, air-exposed reservoirs, multiple different materials and their interfaces requires further investigation and development of the flow simulation methods that can comprehensively include all variables affecting the actual flow and thus, to estimate the flow conditions with a reasonable margin of error.

We were able to discover suitable medium volumes for the gravity-driven flow as we used spatial vascular network formation as a biological indicator demonstrating the combined height of the compartments. We examined GFP-to-RFP signal height after applying medium as Approaches 1–5. In Approach 5, in which all reservoirs were filled maximally, and in Approach 4, in which the upper compartment reservoir was filled to maximum, we detected a drastic decrease of the GFP-to-RFP signal height. We suggest that the generated hydrostatic pressure together with the occurring medium flow disrupted the three-dimensionality of the hydrogel making these flow conditions suboptimal for further examination. However, in *asymmetric-side-to-center*, *symmetric side-to-center* and *symmetric center-to-side* conditions we showed that the suggested flow conditions allowed the medium flow to occur across the cell culture compartments. Simultaneously, these flow conditions maintained the three-dimensionality as we showed only a minor decrease on the examined mean height of the GFP-to-RFP vasculature and verified the stability of the vascular network formation throughout the culture period. Thus, we continued further comparisons in the GFP-RFP-vascular network development with the three flow conditions.

By adjusting the direction and quantity of the medium flow, we demonstrate that the gravity-driven flow enhances the formation of vascular networks. Due to the small sample size, we did not reach statistical significance with our findings, yet by comparing median values of the vessel length we could verify that both the *asymmetric side-to-center* condition and the *symmetric center-to-side* condition showed considerably longer, 1.9-fold of total network length than in the *symmetric side-to-center *condition. It has been shown previously that the developing vasculature benefits from the presence of interstitial flow by inhibiting vessel regression^7,34^. Our results coupled with the results cited above, suggest that the flow provided by the present *symmetric side-to-center* condition is suboptimal as we showed decreased vessel length together with the reduced vascular volume compared to the other flow conditions. Still, medians of the total vessel length are shown to be similar between *asymmetric side-to-center* and *symmetric center-to-side* conditions that might indicate the likeness of the occurring medium flow.

To verify observed differences between GFP-HUVEC distribution in *asymmetric side-to-center* condition, we showed significant difference in GFP-HUVEC distribution around the open top area indicating notable medium flow across the lower cell culture compartment. Previously, Bachmann et al. (2018) highlighted that great interstitial flow may compromise endothelial cell viability in long-term cultures as the fluid flow elutes paracrine, angiogenic molecules and eliminates appropriate cell signaling. This finding is consistent with our simulated, initial pressure distribution facing the fibrin-embedded GFP-vasculature in the lower cell culture compartment and the decreased vascular volume in the *asymmetric side-to-center* condition as great medium flow may impair vessel formation. Still, further experiments are needed to quantitatively investigate and characterize the spatiotemporal fluid flow to determine occurring flow velocities.

Recent studies have demonstrated the feasibility to generate 3D vascular network *in vitro* on microfluidic devices^7,12,25,28,34,35,43–45^, yet the reported vessel diameters failed to recapitulate the typical, narrow capillary diameter in vivo^46^. Here, we report in vivo-relevant median vessel diameter of 12–14 μm in all the studied flow conditions. The resulting diameter range is also in line with previous findings of fibrin-embedded ASC-supported vasculature in vitro^19^. Remarkably, the measured vessel length values are in line with previously reported in vivo values for highly vascularized cerebral cortex in humans^47^.

We verified the openness of vasculature by demonstrating continuous, lumenized vessels in the lower cell culture compartment and examined whether the GFP- and RFP-vasculatures will merge with each other. Confocal imaging revealed prominent migration of RFP-vasculature into the lower culture compartment. We observed great interconnectivity of GFP-RFP-vasculature as abundant co-localization of GFP- and RFP-signals. The existence of vessels with both fluorescent signals indicate that the formed vasculature originates from both compartments and can branch and fuse between the compartments. As we first pipetted fibrin-embedded RFP-HUVECs together with ASCs and subsequentially the GFP-HUVEC-ASC co-culture on top, our results suggest that all RFP-vessels occurring outside the upper cell culture compartment have reached the lower culture compartment solely via migration. Taken together, these results suggest that the formed vascular network in the developed microfluidic device originates from both cell culture compartments and truly recapitulates a physiologically relevant vascular density with an appropriate vessel diameter.

To conclude, we report a novel, barrier-free open-top microfluidic device that is applicable for generating two distinct 3D co-cultures under the desired flow condition and allows cells to fluently migrate between the culture compartments. With a phaseguide restricting the cell-laden hydrogel, we maximized the interaction between the cell culture compartments and the flowing medium. We characterized three different flow profiles resulting in distinct networks characteristics. All the formed vasculatures were shown to have lumenized vessels with in vivo-relevant vessel diameter and length. The structural association of two distinct, ASC-supported vasculatures demonstrates the feasibility of the device for generating more realistic, organotypic cultures of human tissues. Importantly, the accessible upper cell culture compartment allows temporal control in terms of cell plating that is a challenge in microfluidic devices with closed cell culture chambers. Due to the universal design of the microfluidic device, the developed platform is adaptable for generating various experimental models of vascularized co-cultures and guide strategies to produce vascularized tissue models *in vitro.*

## Electronic supplementary material

Below is the link to the electronic supplementary material.

Supplementary Material 1

Supplementary Material 2

Supplementary Material 3

## Data Availability

The image post-processing parameters and data sets generated and analyzed for this study are available from the corresponding author on request.
