# Supplementary material for: Barrier-free, open-top microfluidic chip for generating two distinct, interconnected 3D microvascular networks
Source: Sci Rep. 2024 Oct 2;14:22916. doi: 10.1038/s41598-024-74493-3 (PMC11447027; doi:10.1038/s41598-024-74493-3)
Supplement: Supplementary file 1 — Supplementary Material 1 [file 41598_2024_74493_MOESM1_ESM.pdf]

Supplementary data sheet

Barrier-free, open-top microfluidic chip for generating two distinct, interconnected 3D microvascular networks

Alma Yrjänäinen[1,2], Elina Mesiä[3], Ella Lampela[1,2], Joose Kreutzer[3], Jorma Vihinen[4], Kaisa Tornberg[3], Hanna Vuorenpää[1,2], Susanna Miettinen[1,2], Pasi Kallio[3] and Antti-Juhana Mäki[3]

[1]Adult Stem Cell Research Group, Faculty of Medicine and Health Technology, Tampere University, Tampere, Pirkanmaa, Finland

[2]Tays Research Services, Wellbeing Services County of Pirkanmaa, Tampere University Hospital, Tampere, Pirkanmaa, Finland

[3]Micro- and Nanosystems Research Group, Faculty of Medicine and Health Technology, Tampere University, Tampere, Pirkanmaa, Finland

[4]Faculty of Engineering and Natural Sciences, Tampere University, Tampere, Pirkanmaa, Finland

Corresponding author: alma.yrjanainen@tuni.fi

Table S1. Quantitative values for the studied parameters: vascular volume (VOL_, unit V-% i.e. vascular volume per total volume of ROI), diameter (DIA_, unit µm) and total vessel length (L_, unit µm) in each flow condition ($n_{\textbf{asymmetric side-to-center}}$=10, $n_{\textit{symmetric center-to-side}}$=10, $n_{\text{symmetric side-to-center}}$=9). Values are retrieved from Imaris software based on created surface and filament of fluorescently tagged GFP-HUVECs.

VOL_Asymmetric **side-to-center**	VOL_Symmetric *center-to-side*	VOL_Symmetric side-to-center	DIA_Asymmetric **side-to-center**	DIA_Symmetric *center-to-side*	DIA_Symmetric side-to-center	L_Asymmetric **side-to-center**	L_Symmetric *center-to-side*	L_Symmetric side-to-center
2,1	1,4	1,2	10,7	10,7	8,4	1819	1117	1207
2,3	1,4	1,5	10,7	11,1	11,1	1941	1974	1214
2,5	2,1	1,8	11,3	12,2	12,3	2171	2044	1243
2,8	3,7	1,8	11,6	14,0	12,7	2310	2362	1414
3,0	4,6	2,4	11,7	14,3	12,7	2416	2709	1427
3,5	4,7	2,5	11,9	14,4	13,1	3127	2713	1857
4,0	4,8	3,4	12,9	14,7	13,8	3410	3001	2178
4,4	5,3	5,2	13,4	15,8	15,9	3599	3106	3018
5,7	5,8	5,8	14,3	16,7	16,8	4318	3217	3550
6,4	9,9		14,9	17,5		5407	4554	

Table S2. Correlation analysis matrices for the quantified vascular network parameter values (Table S2) ($n_{asymmetric\ side\text{-}to\text{-}center}$=10, $n_{symmetric\ center\text{-}to\text{-}side}$=10, $n_{symmetric\ side\text{-}to\text{-}center}$=9), vascular volume (VOL_ i.e. V-% of total volume of ROI), diameter (DIA_) and total vessel length (L_), as a heatmap with respective r values. Correlation was studied within each flow condition with GraphPad Prism 9.

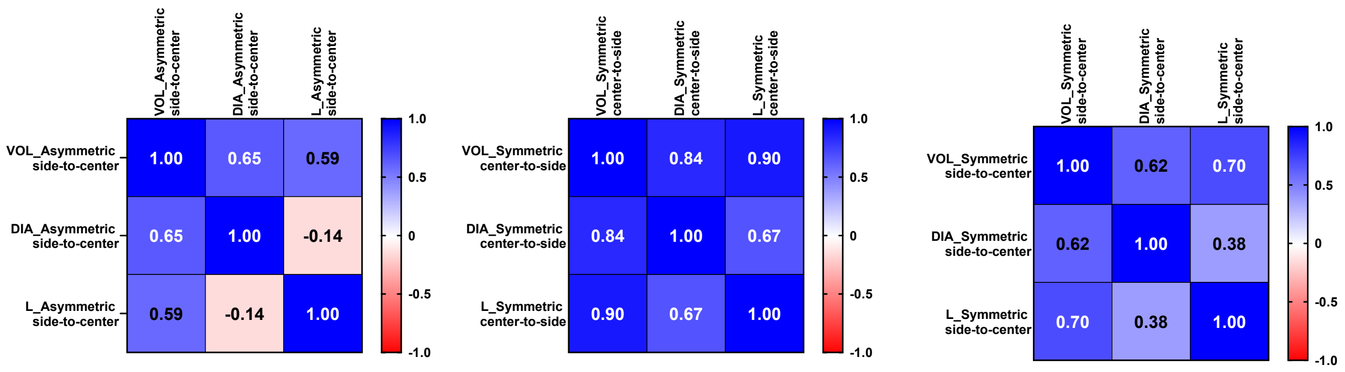

Parameter 1	Parameter 2	r value	p value	Significancy
VOL_Asymmetric side-to-center	DIA_Asymmetric side-to-center	0,65	0,049	*
VOL_Asymmetric side-to-center	L_Asymmetric side-to-center	0,59	0,081	ns
VOL_Symmetric center-to-side	DIA_Symmetric center-to-side	0,84	0,0037	**
VOL_Symmetric center-to-side	L_Symmetric center-to-side	0,90	0,00081	***
DIA_Symmetric center-to-side	L_Symmetric center-to-side	0,67	0,039	*
VOL_Symmetric side-to-center	DIA_Symmetric side-to-center	0,62	0,086	ns
VOL_Symmetric side-to-center	L_Symmetric side-to-center	0,70	0,043	*

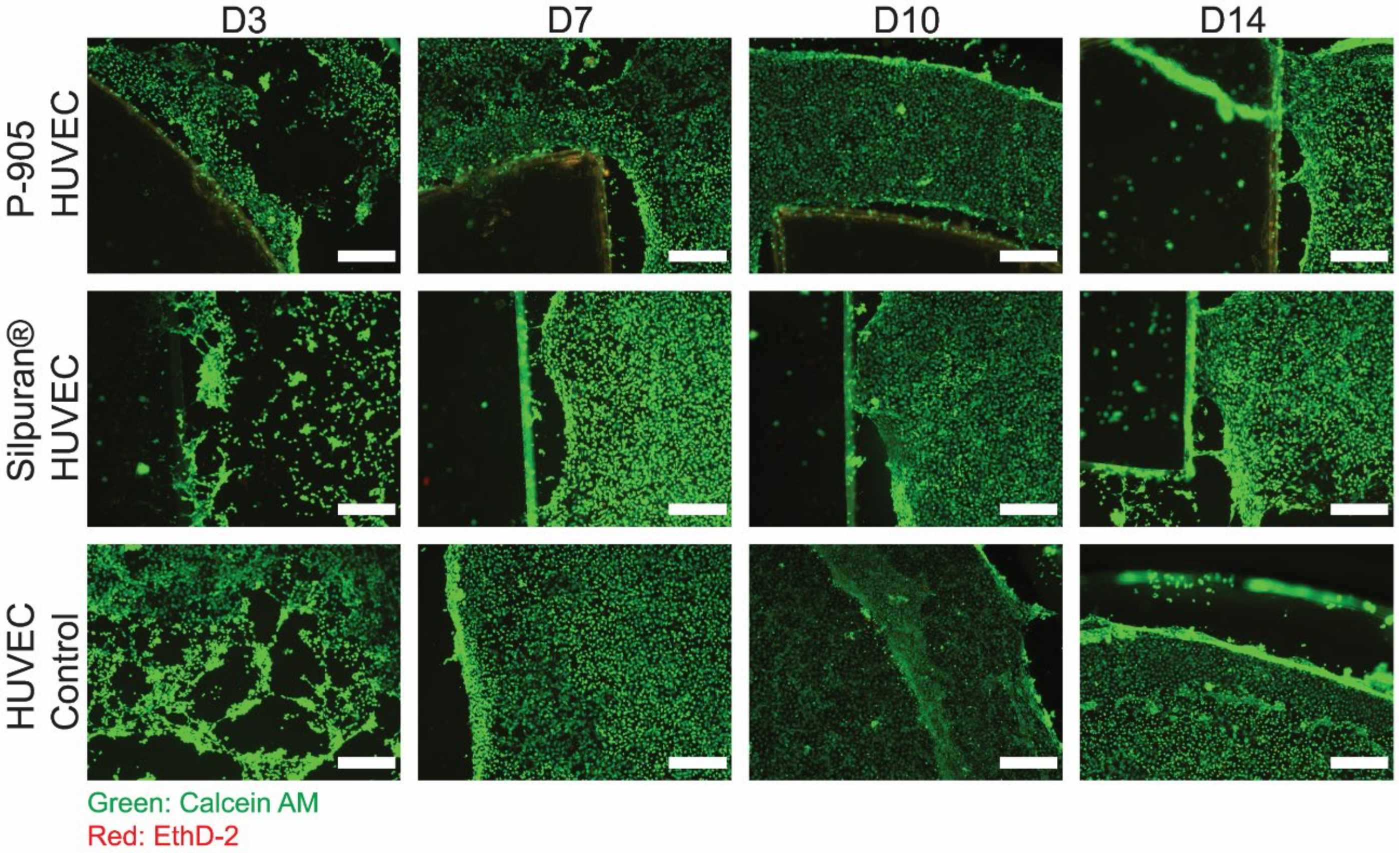

Fig S1. Cytocompatibility examination for the used materials in microfluidic channels, P-905 tape, and in hydrogel guide, Silpuran® film. Primary human umbilical vein endothelial cells (HUVECs) were sourced from umbilical cord samples from Tampere University Hospital (Regional Ethics Committee of Tampere University Hospital, supportive ethics statement R13019). Prior cytocompatibility evaluation, isolated HUVECs were thawed and cultured in Endothelial Cell Growth Medium-2 (EGM-2) (Lonza, Basel, Switzerland). The P-905 tape and Silpuran® film samples were prepared according to the chip fabrication protocol and inserted to the bottom of the 48-wellplate. Then, HUVECs were plated 20 000 cells / cm^2 onto tested materials (Huttala et al. 2015) and cultured for 14 days. EGM-2 media was changed every 2 days and HUVECs were imaged with an inverted widefield fluorescence microscope (Olympus IX, Tokyo, Japan). The viability of HUVECs was assessed through qualitative inspection using LIVE / DEAD® Viability / Cytotoxicity Kit for mammalian cells (Invitrogen, Waltham, USA). The assay was performed on cell culture days 3, 7, 10, and 14. EGM-2 was aspirated from the culture wells and the cells were washed with 1 X Dulbecco's phosphate-buffered saline (1XDPBS) (17-512F, Lonza). Then, 400 µl of LIVE / DEAD® solution was added to each well and incubated on a rocker covered by light. Finally, the samples were imaged using 488nm and 568nm filters and 4x objective. Fluorescent images of LIVE / DEAD™ stained HUVECs demonstrated comprehensive viability in the presence of both chip materials through the entire experiment of 14 days. Scale bar 500 µm.

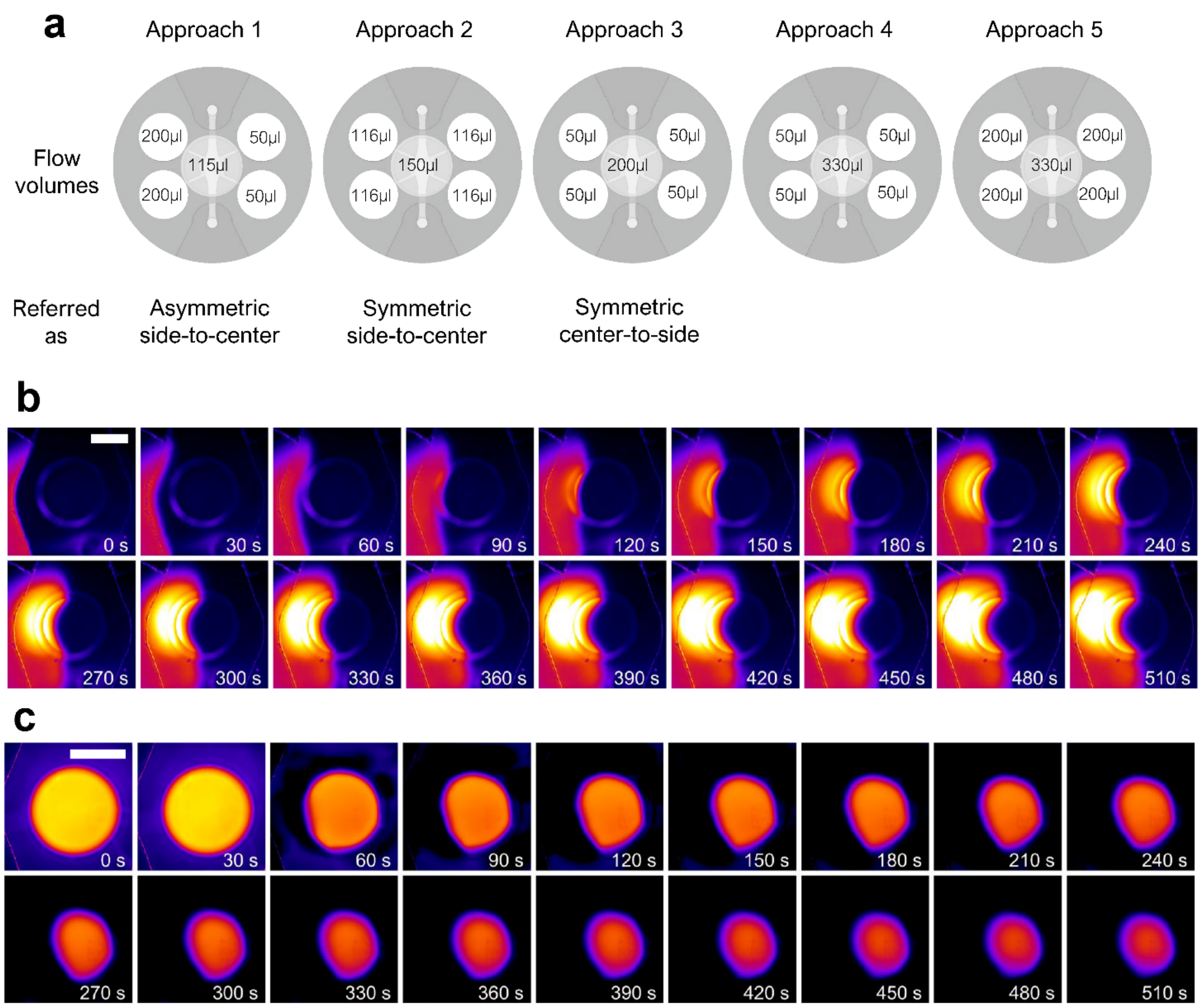

Figure S2 Flow conditions in the cell culture compartments. (A) Studied flow profiles for microvascular network formation. Approaches 1,3 and 5 were further selected for experimental work and named as asymmetric side-to-center, symmetric center-to-side and symmetric side-to-center, respectively. (B) Increasing dextran flow front was imaged in Approach 1 to demonstrate occurring flow across the culture compartments and decreasing dextran intensity in Approach 5 depicting the flow penetrating the culture compartments. Scale bars 1 mm.

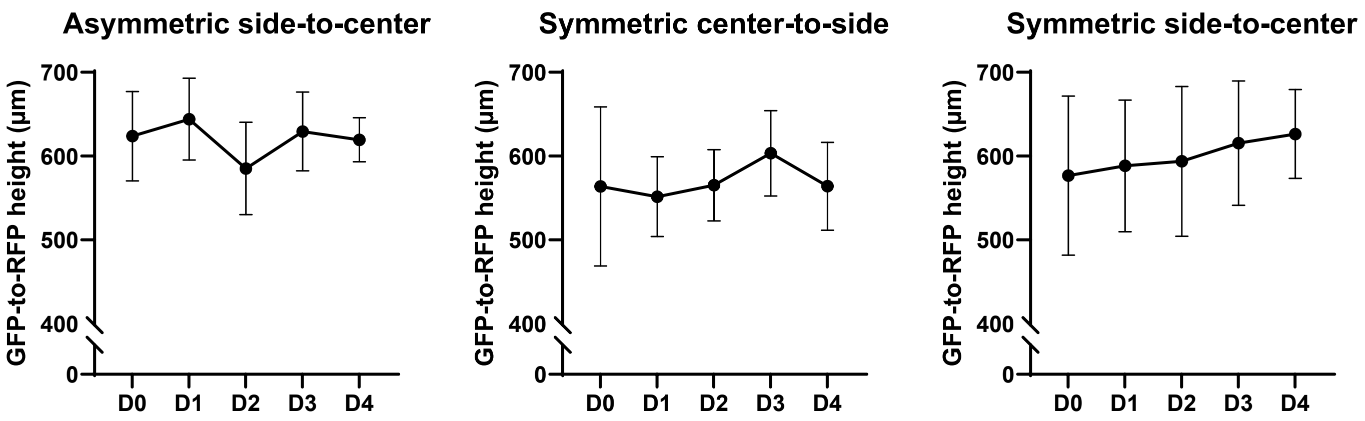

Fig S3. GFP-to-RFP -height in z-direction during cell culture D0-D4. All flow conditions maintained overall hydrogel integrity. Positive change in GFP-to-RFP height is explained by vessel formation upwards.

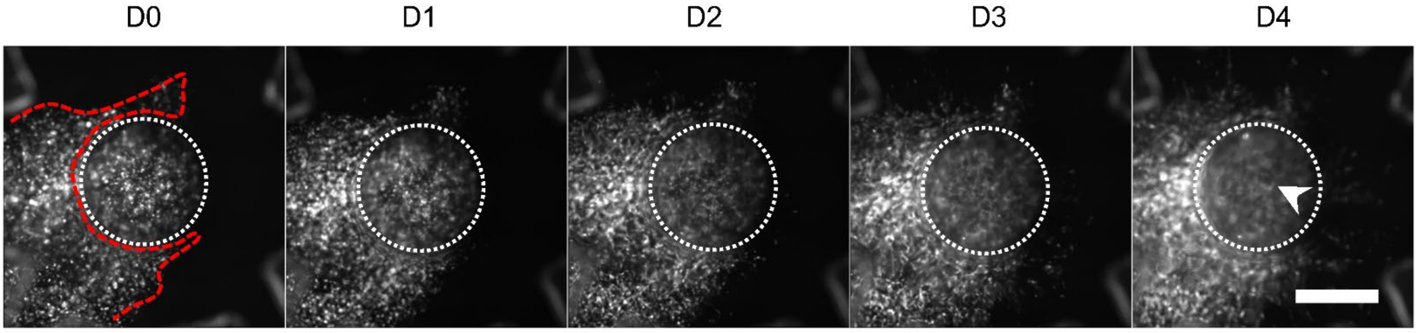

Figure S4 Daily development of RFP-HUVEC vascular network on open top chip. The detected RFP-signal shows vessel formation (white arrows) within the upper cell culture compartment (dashed, white circle). Detection height >400µm from the bottom glass. Vessel formation around the upper cell culture compartment shows the excess cell-laden fibrinogen gelated arbitrarily onto top glass due to plating (dashed, red line). Scale bar 1 mm.

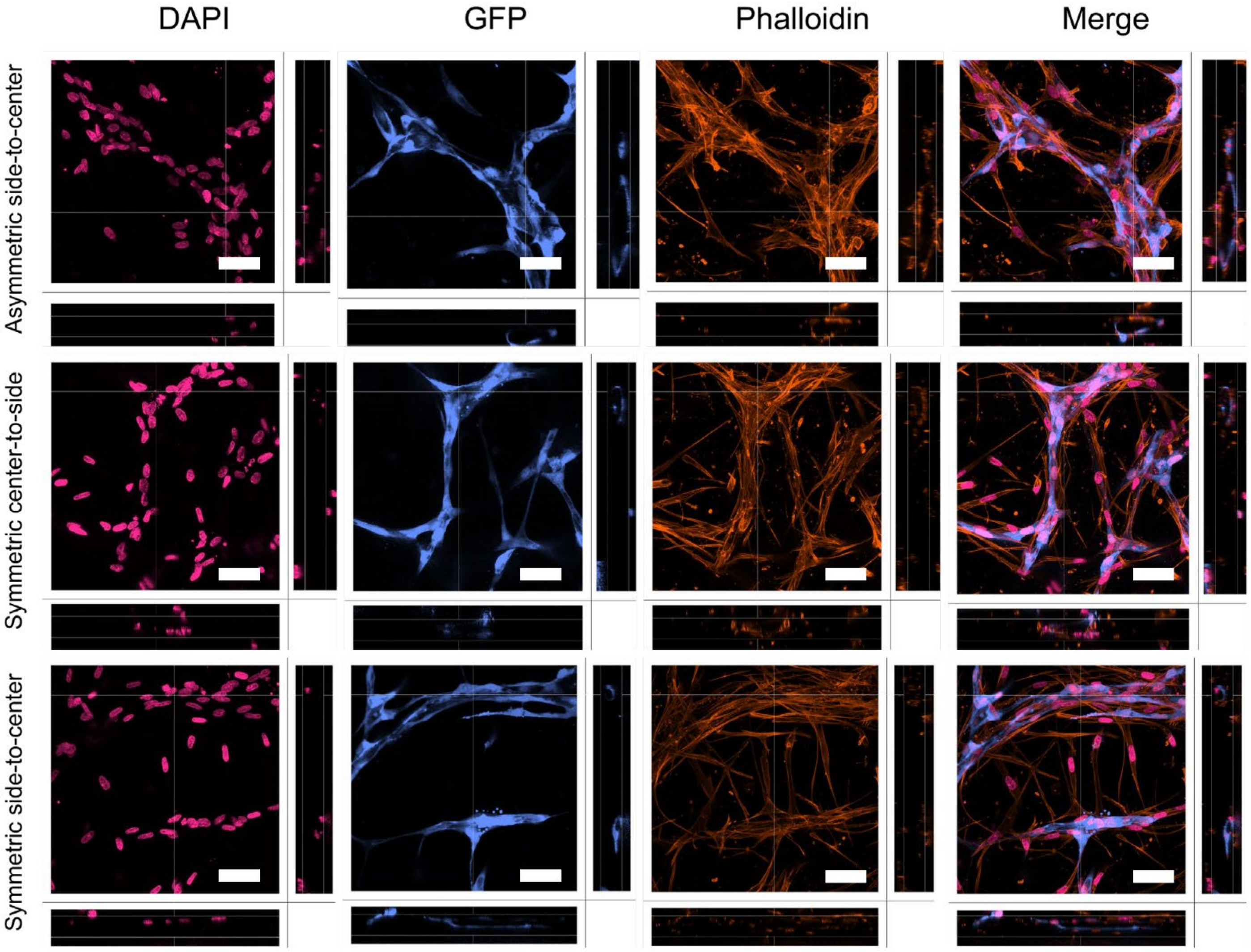

Fig. S5 Individual channels for all flow conditions showing lumenized vessels with *in vivo*-relevant diameter. Orthogonal view depicts the x- and y- projections of the hollow vessel showing continuous endothelial cell lining (GFP, cyan), actin skeletons of GFP-HUVECs and ASCs (phalloidin, orange) and nuclei (DAPI, magenta). Scale bars 50 µm.

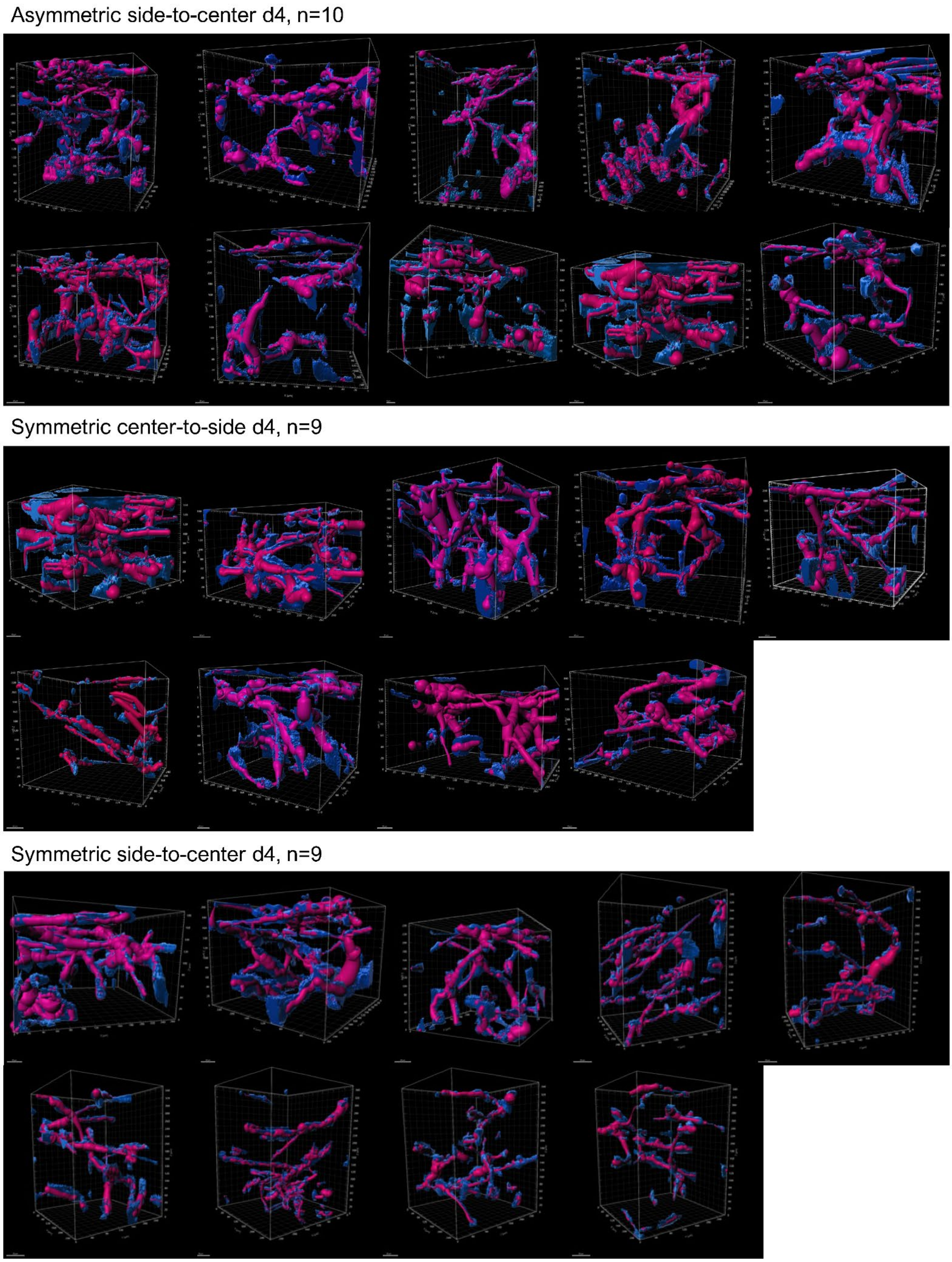

Fig. S6 Representative images of the processed ROIs for vascular network characterization in each flow condition: asymmetric side-to-center (n=10), symmetric center-to-side (n=9) and symmetric side-to-center (n=9). GFP-HUVEC-based signal was used to create a quantifiable surface (blue) and a filament (pink) for statistical examination of vascular volume and filament (pink) and total vessel length per ROI. Scale provided per each image.

Text file S1. Imaris parameters for creating the GFP-surface objects.

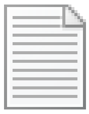

Surfaces Creation Parameters.txt

Text file S2. Imaris parameters for creating the GFP-filament objects from masked GFP-channel. GFP-surface (S5) was used to mask the GFP-channel (on which Normalize layers and Linear stretching were applied), setting voxel values inside the surface to 0 and outside the surface to 3000.

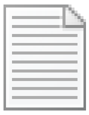

Filament Creation Parameters.txt
